# Patient-Reported Prevalence of Non-motor Symptoms Is Low in Adult Patients Suffering From 5q Spinal Muscular Atrophy

**DOI:** 10.3389/fneur.2019.01098

**Published:** 2019-11-01

**Authors:** René Günther, Claudia Diana Wurster, Isabell Cordts, Jan Christoph Koch, Christoph Kamm, Daniel Petzold, Elisa Aust, Marcus Deschauer, Paul Lingor, Albert Christian Ludolph, Andreas Hermann

**Affiliations:** ^1^Department of Neurology, Technische Universität Dresden, Dresden, Germany; ^2^German Center for Neurodegenerative Diseases Dresden, Dresden, Germany; ^3^Department of Neurology, Ulm University, Ulm, Germany; ^4^Department of Neurology, Technical University of Munich, School of Medicine, Klinikum rechts der Isar, Munich, Germany; ^5^Department of Neurology, University Medical Center Göttingen, Göttingen, Germany; ^6^Department of Neurology, University Medical Center Rostock, University of Rostock, Rostock, Germany; ^7^German Center for Neurodegenerative Diseases Ulm, Ulm, Germany; ^8^Translational Neurodegeneration Section “Albrecht-Kossel”, Department of Neurology, University Medical Center Rostock, University of Rostock, Rostock, Germany; ^9^German Center for Neurodegenerative Diseases Rostock, Rostock, Germany

**Keywords:** spinal muscular atrophy (SMA), motor neuron disease (MND), multisystem disorder, non-motor symptoms, NMSQuest

## Abstract

**Background:** 5q spinal muscular atrophy (SMA) is an autosomal recessive lower motoneuron disease caused by deletion or mutations in the survival motor neuron 1 gene (*SMN1*) which results in reduced expression of full-length SMN protein. The main symptoms are caused by spinal motor neuron demise leading to muscle atrophy, and medical care mostly refers to motor symptoms. However, new insights of recent studies in severe SMA type I revealed disease involvement of several non-motor regions, for example cardiac, vascular, sensory nerve involvement, and thalamic lesions. Non-motor symptoms (NMS) were previously described in many neurodegenerative diseases i.e., Parkinson's disease and, importantly, also amyotrophic lateral sclerosis.

**Methods:** We screened for NMS in 70 adult patients with SMA type II (SMAII) and type III (SMAIII) and 59 age/sex-matched healthy controls (controls) in a multicenter cross-sectional study including 5 different centers with specialized expertise in medical health care of motoneuron diseases. We used a self-rating questionnaire including 30 different items of gastrointestinal, autonomic, neuropsychiatric, and sleep complaints [NMS questionnaire (NMSQuest)], which is a validated tool in Parkinson's disease.

**Results:** Total NMS burden was low in adult SMA (median: 3 items) and not significantly different compared to controls (median: 2 items). Total NMS of SMA patients did not correlate with disease severity scores. However, the items “swallowing difficulties,” “falling,” and particularly “swelling legs” were significantly more frequently reported in SMA. Neuropsychiatric symptoms were reported in a frequency comparable to controls and were not significantly increased in SMA.

**Conclusion:** Patient-reported prevalence of NMS in adult SMA was low, which does not argue for a clinically relevant multisystemic disorder in SMAII/III. Importantly, adult SMA patients do not seem to suffer more frequently from symptoms of depression or adaptive disorders compared to controls. Our results yield novel information on previously underreported symptoms and will help to improve the medical guidance of these patients.

## Introduction

5q-associated spinal muscular atrophy (SMA) is one of the most common neuromuscular diseases in childhood and is caused by homozygous deletion or less frequently other mutations in the survival motor neuron 1 gene (*SMN1*). The protein encoded by this gene, SMN, is ubiquitously expressed and eukaryotic cells are not viable without SMN protein. Despite the homozygous deletion in *SMN1*, patients with SMA are viable because of the existence of insufficient intrachromosomal duplications of *SMN1*, the *SMN2* gene, centromeric to *SMN1*. However, a base pair substitution in exon 7 and the intron silence sequence in intron 7 of *SMN2* lead to an altered splicing, and consequently exon 7 is missing in ~90% of final mRNAs. Skipping of exon 7 leads to an unstable, non-functional SMN protein, which is immediately degraded (1). The clinical phenotype of the disease is more or less devastating, depending largely on the number of *SMN2* copies (2). Although SMN is expressed in all tissues, lower amounts of SMN primarily affects lower motoneurons (MN) leading to muscle weakness and muscle atrophy (3). SMA was originally thought to be a pure motoneuron disease (MND), but recent evidence suggests a multisystem involvement (4). The preferential involvement of proximal muscle groups also suggests differences in vulnerability against SMN deficiency of different MN pools. In SMA animal models it has been demonstrated that distinct motor units have different susceptibility to the lack of SMN (5, 6). Additionally, we recently showed that denervation of hand muscles in SMA patients is not equally distributed. In contrast to amyotrophic lateral sclerosis (ALS), adult SMA type 2 (SMAII), and 3 (SMAIII) patients presented with a typical denervation pattern of the hand muscles (reversed split hand) (7). SMN is ubiquitously expressed, and SMN levels are reduced in different tissues in SMA animal models including spinal cord, brain, kidney, liver, heart and muscle (8). A very recent study on health insurance data revealed numerous non-neuromuscular phenotypes which had been diagnosed in the cardiovascular, gastrointestinal, metabolic, reproductive, and skeletal systems in pre-diagnostic SMA (9). Fitting to that, neuropathological studies revealed widespread neuronal degeneration also in dorsal root ganglia, thalamus, cerebral cortex, basal ganglia, pigmented nuclei, brainstem and cerebellum in severely affected SMA type 1 patients (10, 11). Studies on sural nerve biopsies revealed sensory nerve pathology in SMA type 1 patients, but not in type 2 or 3 (12) and cases of vascular necrosis/vascular thrombosis were reported in SMA type 1 patients (13, 14). While congenital heart disease is a phenomenon of SMA type 1 (15), involvement of the heart revealed by electrocardiography seems to be evident in all types of SMA (16). Recently, dysfunction of energy metabolism and increased levels of leptin were found in SMA type 1 to 3 patients, which was associated with disease severity and decreased motor function. It was hypothesized that neuronal degradation of hypothalamic cells or an increase in fat content by muscle remodeling could be the cause of the highly prevalent hyperleptinemia in SMA patients (17).

To our knowledge, a systematic evaluation of the prevalence and significance of non-motor symptoms (NMS) in adult SMA has not yet been reported. The aim of this study was therefore to assess the NMS burden in adult patients with SMAII and SMAIII with the NMS questionnaire (NMSQuest).

## Methods

### Participants

We analyzed data from 70 genetically confirmed SMAII and SMAIII as well as 59 healthy controls (for details see Table 1). We only included healthy subjects, if no physical or psychological disabilities/diagnoses were reported, none of them suffered from a chronic devastating disease. Data were collected from five different centers of expertise in motoneuron diseases: Technische Universität Dresden (Germany), Ulm University (Germany), Technical University of Munich (Germany), University of Rostock (Germany) and University Medicine Göttingen (Germany). All patients and controls gave their informed consent and study approval was obtained by the local ethics committees (EK393122012; A 2014-0021; EK19/12, 2012; P001660A; 2019-0054, 10/2/17).

**Table 1:** Demographic and clinical characteristics of the study populations.

	**Controls**	**SMA**
Number	59	70
Ratio of female (%)	57.9	45.7
Age (yr)	35.3 ± 9.2	35.9 ± 10.9
Subtypes (n)	–	SMAII (27) SMAIII (43)
Nusinersen treatment (yes/no)	0/59	40/30
Wheelchair-bound/ambulatory	0/59	49/21
HFMSE	–	19.7 ± 21.7
RULM	–	19.5 ± 13.2
ALSFRS-R	–	31.1 ± 8.6

### Assessments

The NMSQuest is a 30-item self-completed questionnaire featuring responses as “yes” and “no” to each item and was originally designed and validated for Parkinson's disease patients (18–20). It is a widely used screening questionnaire also used in amyotrophic lateral sclerosis (ALS) and cervical dystonia (21–23). Its 30 items were termed according to the publication of Chadhuri et al. (18) and can be grouped into 9 domains (digestive, urinary, memory, perceptions, mood, sex, cardiovascular, sleep, and miscellaneous). “Total NMS score” was defined by the sum of all positive (“yes”) answers of the 30 items. We additionally recorded data of age, gender, revised ALS-Functional-Rating Scale (ALSFRS-R), Hammersmith Functional Motor Scale Expanded (HFMSE) and Revised Upper Limb Module (RULM).

### Statistical Analysis

As the samples were not normally distributed according to the Kolmogorov-Smirnov test, the statistical comparisons of data between groups were performed using the non-parametric Mann–Whitney *U*-test (MWU) for total NMS score and age. Pearson's chi-squared test (χ2) was carried out for a comparison of gender distribution and for a comparison of the proportion of “yes” and “no” responses between patients and controls for each single item. Spearman rank correlation coefficients were used to examine correlations between total NMS score and clinical characteristics with a correlation coefficient of rho <0.3 considered as a weak, rho = 0.3–0.59 a moderate, and rho ≥0.6 a strong correlation. Data were analyzed using the software SPSS 21.0 (SPSS Inc., Chicago, IL, USA) and Statistica 13.2 [StatSoft (Europe) GmbH, Hamburg, Germany]. If not mentioned otherwise, all data are displayed as means ± standard deviation (SD). Significance level was set at *p* < 0.05. Correction for multiple testing was not applied as the study is merely exploratory, hypothesis-generating, thus a previous hypothesis was not proposed.

## Results

### Demographic and Clinical Characteristics

NMSQuest data from 70 adult SMAII and SMAIII patients and 59 age/sex matched controls were analyzed and compared. Demographic and clinical characteristics of study populations are shown in Table 1. 57.1% of the patients were under nusinersen treatment to the time point of survey. Thirty percent of patients were ambulatory and 70% full-day wheelchair-bound. Except for the item “sex difficulty” and “changes of sex drive,” which was not answered by 3 patients and 1 control, all other questions were fully answered of all subjects. Two of the controls reported extremely high numbers of NMS (18 and 23 items, respectively) and were classified as extreme values and therefore removed from the analysis. SMA and control group did not differ significantly in age (*p* = 0.85) or gender (*p* = 0.17). Most patients were severely affected by the disease (HFMSE: 19.7 ± 21.7; RULM 19.5 ± 13.2, ALSFRS-R: 31.1 ± 8.6).

### NMS in SMA Patients

**Figure 1:**
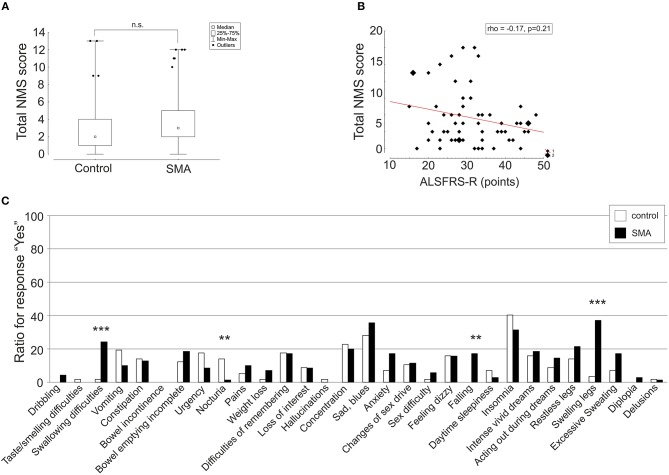
Patient-reported prevalence of non-motor symptoms (NMS) in adult 5q spinal muscular atrophy (SMA). Total NMS score **(A)** depicted as box plot and ratio of response “yes” of single items **(C)** depicted as histograms were compared between SMA (black) and controls (controls) (white). Correlation of total NMS score and ALSFRS-R is shown as scatterplot **(B)**. Spearman rank correlation coefficient (rho). ^**^p < 0.01, ^***^p < 0.001, n.s., not significant.

Total NMS were not significantly different between SMA and controls (*p* = 0.07) (Figure 1A). The sum of NMS ranged from 0 to 12 items in SMA patients with a median of 3 items and in controls from 0 to 13 items with a median of 2 items. Total NMS score was not significant different (*p* = 0.45) between nusinersen treated patients (*n* = 40, median 3, range 0 to 12 items) and untreated patients (*n* = 30, median 2.5, range 0 to 12 items). Total NMS of SMA patients did not correlate with disease severity scores (HFMSE rho = −0.02, *p* = 0.87, *N* = 65; RULM rho = −0.06, *p* = 0.61, *N* = 66; ALSFRS-R rho = −0.17, *p* = 0.21, *N* = 59) (Figure 1B). However, SMA patients complained about the items “swallowing difficulties,” “falling,” and “swelling legs” significantly more frequently compared to controls (Figure 1C, for details see Table 2). “Excessive sweating” was 10% more frequently reported in the SMA group, but was not significantly different to controls (*p* = 0.09). SMA patients who reported the item “swallowing difficulties” had significant lower scores on ALSFRS-R (Yes: 24.1 ± 4.6, No: 32.8 ± 8.6, *p* < 0.0001), RULM (Yes: 6.2 ± 6.9, No: 20.2 ± 13.2, *p* = 0.016) and HFMSE (Yes: 2.4 ± 4.0, No: 19.4 ± 21.5, *p* = 0.042) compared to SMA patients who did not complain for “swallowing difficulties.” HFMSE (Yes: 8.7 ± 11.8, No: 21.5 ± 23.5, *p* = 0.038), but not ALSFRS-R (Yes: 28.4 ± 7.0, No: 33.03 ± 9.2, *p* = 0.087) or RULM (Yes: 14.9 ± 12.6, No: 19.5 ± 13.8, *p* = 0.077) were significantly lower in SMA patients who reported “swelling legs” compared to SMA patients who did not. SMA patients who reported “swelling legs” were significantly more often full-day wheelchair-bound (Yes: 22 of 26 were wheelchair-bound, No: 27 of 44 were wheelchair-bound, *p* = 0.04). “Falling” was reported from SMA patients with higher scores in HFMSE (Yes: 43.0 ± 15.6, No: 11.8 ± 17.7, *p* < 0.001), ALSFRS-R (Yes: 41.4 ± 4.6, No: 29.4 ± 8.0, <0.001) and RULM (Yes: 31.5 ± 7.8, No: 15.3 ± 12.8, *p* < 0.001). “Falling” was significantly more often reported from ambulatory patients (Yes: 10 of 12 patients were ambulatory, No: 47 of 58 were wheelchair-bound, *p* < 0.001). ALSFRS-R, HFMSE und RULM were not significantly different between SMA patients who reported “excessive sweating” and patients who did not.

**Table 2:** Comparison of NMS between controls and SMA patients.

	**Distribution of response “yes” in percentages**		**Chi-squared-test**
	**controls**	**SMA**	**for yes–no**
Dribbling	0	4.3	0.114
Taste/smelling difficulties	1.8	0	0.266
Swallowing difficulties^***^	1.8	24.3	0.000
Vomiting	19.3	10.0	0.135
Constipation	14.0	12.9	0.846
Bowel incontinence	0	0	n.c.
Bowel emptying	12.3	18.6	0.333
Urgency	17.5	8.6	0.130
Nocturia^**^	14.0	1.4	0.006
Pains	5.3	10	0.324
Weight loss	1.8	7.1	0.155
Difficulties of remembering	17.5	17.1	0.953
Loss of interest	8.8	8.6	0.968
Hallucinations	1.8	0.0	0.266
Concentration difficulties	22.8	20.0	0.701
Sad, blues	28.1	35.7	0.359
Anxiety	7.0	17.1	0.087
Changes of sex drive	10.5	11.4	0.872
Sex difficulty	1.8	5.7	0.262
Felling dizzy	15.8	15.7	0.991
Falling ^**^	0	17.1	0.001
Daytime sleepiness	7.0	2.9	0.272
Insomnia	40.4	31.4	0.296
Intense vivid dreams	15.8	18.6	0.680
Acting out during dreams	8.8	14.5	0.324
Restless legs	14.0	21.4	0.282
Swelling legs^***^	3.5	37.1	0.000
Excessive Sweating	7.0	17.1	0.087
Diplopia	0	2.9	0.198
Delusions	1.8	1.4	0.883

Significantly different items are labeled with

** *p < 0.01*,

*** *p < 0.001; n.c., non-calculable*.

Comparison of SMAII and SMAIII revealed no significant differences in total NMS score (*p* = 0.99). The sum of NMS ranged from 0 to 12 in SMA patients with median 3 in both groups. Significant differences were only found for two items. The item “falling” was more frequently reported by SMAIII patients, however it has to be taken into account that SMAII are not ambulatory (per definition) and therefore have generally a lower risk to fall. This is highlighted by the fact that the item falling is also strongly depending on the severity score HFMSE. The second difference between SMAII and SMAIII patients was the item “acting out during dreams,” which was more frequently reported by SMAII, however still not significantly different to controls (*p* = 0.06). All other items are not significantly different between SMAII and SMAIII (see Table 3).

**Table 3:** Comparison of NMS between SMAII and SMAIII.

	**Distribution of response “yes” in percentages**		**Chi-squared-test**
	**SMAII**	**SMAIII**	**for yes–no**
Dribbling	7.4	2.3	0.307
Taste/smelling difficulties	0	0	n.c.
Swallowing difficulties	29.6	20.9	0.409
Vomiting	11.1	9.3	0.806
Constipation	11.1	14.0	0.729
Bowel incontinence	0	0	n.c.
Bowel emptying	22.2	16.3	0.534
Urgency	7.4	9.3	0.783
Nocturia	0	2.3	0.425
Pains	11.1	9.3	0.806
Weight loss	7.4	7.0	0.946
Difficulties of remembering	18.5	16.3	0.809
Loss of interest	7.4	9.3	0.783
Hallucinations	0	0	n.c.
Concentration difficulties	22.2	18.6	0.713
Sad, blues	40.7	32.6	0.487
Anxiety	25.9	11.6	0.122
Changes of sex drive	18.5	7.0	0.140
Sex difficulty	7.4	4.7	0.629
Felling dizzy	22.2	11.6	0.236
Falling^*^	3.7	25.6	0.018
Daytime sleepiness	0	4.7	0.256
Insomnia	25.9	34.9	0.432
Intense vivid dreams	26.0	14.0	0.210
Acting out during dreams^*^	25.9	7.1	0.031
Restless legs	14.8	25.6	0.285
Swelling legs	37.0	37.2	0.988
Excessive Sweating	14.8	18.6	0.682
Diplopia	0	4.7	0.256
Delusions	0	2.3	0.425

Significantly different items are labeled with

* *p < 0.05; n.c., non-calculable*.

## Discussion

SMA pathophysiology is caused by homozygous deletion or mutations in the *SMN1* gene, which encodes the ubiquitously expressed SMN protein. However, the phenotype of the disease is dominated by lower MN symptoms, therefore the disease is termed “spinal muscular atrophy.” So far, only few studies focused on non-motor involvement in this disease, mainly focused on children with SMA type 1. This is the first study reporting a comprehensive assessment of NMS burden in adult SMA patients.

Patient-reported prevalence of NMS was generally low and, did not—in total—differ significantly from healthy controls and also did not correlate with the severity scores ALSFRS-R, HFMSE and RULM. However, patients in the SMA group complained significantly more frequently about the items “swallowing difficulties,” “falling,” and “swelling legs.” “Swallowing difficulties” were reported by patients who were more severely affected, as indicated by the specific severity scores, as compared to those who were less affected, which may be explained by more prominent bulbar involvement in more severe disease stages. “Falling” was significantly more often complained by patients who were less severely affected by the disease and ambulatory which is explained by the fact that more severely disabled patients are unable to walk and wheelchair-bound and therefore have a lower risk to fall. “Swelling legs” was reported from patients who had lower HFMSE scores. “Swallowing difficulties” and “falling” can be attributed to motor symptoms, but “swelling legs” is highly prevalent and might be a real NMS in SMA. Several reasons could explain this NMS in SMA. It might be a consequence of venous insufficiency due to abnormal posture and/or immobility, as many of the adult patients are fully wheelchair-bound and/or due to decreased muscle strength of the lower limbs with reduced venous muscle pump. Fitting to this hypothesis, patients in this study who complained for “swelling legs” were significantly more frequently full-day wheelchair-bound. On the other hand, it may be related to cardiac insufficiency. Disease involvement of the cardiac muscle has been demonstrated in animal models and patients with severe forms of SMA (15, 24, 25) and there are indirect indications to cardiac alterations reflected by electrocardiographic changes in all SMA types (16). Evaluation of cardiac function is not regularly included in routine health care of these patients. Functional and histological studies are necessary to further study cardiac involvement in adult patients with SMAII and SMAIII.

Neuropsychiatric related complaints like “anxiety,” “loss of interest,” “concentration difficulties,” “feeling dizzy” and “insomnia” were reported in a frequency comparable to healthy controls and were not significantly increased in SMA patients. This may appear surprising, because the disease reduces self-reliance and mobility and often leads to dependency on medical aids and comprehensive medical support. However, it fits to the general impression that most adult SMA patients show a high degree of resilience and no signs of adaptive disorders or depression. This observation is confirmed by a recent published study, where Fischer et al. showed that psychological well-being in adult SMA patients was comparable to that of healthy controls and was unrelated to sociodemographic variables or illness characteristics (26).

Urinary symptoms, including “bowel incontinence,” “bowel emptying incomplete,” “urgency,” and “nocturia,” were only rarely reported by SMA patients. Only a few SMA patients reported “nocturia,” and this was significantly less frequent compared to controls. It could be concluded, that urinary symptoms are not present in these patients. The reason why the item “nocturia” was significantly less frequently reported might be that patients have learned to cope with their motor disabilities, which do not allow them to reach the bathroom in an acceptable time during night.

Comparing groups of patients with SMAII and SMAIII revealed no significant differences in total NMS score. As expected, the item “falling” was significantly more frequent in SMAIII, because all SMAII patients were wheelchair-bound and had therefore a lower risk to fall. The item “acting out during dreams” was more frequent in SMAII, however still not significantly different from controls.

Taken together, patient-reported prevalence of NMS in adult patients with SMA was generally low, arguing for a low perceived burden of other than the motor system. However, “swelling legs” might be a relevant NMS in SMA and its etiology has to be investigated in further studies. Further studies including structured interviews and detailed instrument-based measurements of NMS are warranted to unravel the amount of actual and subclinical NMS in adult SMA.

There are several limitations of this study. First, the screening for NMS was based on a self-rating questionnaire, which was originally designed for Parkinson's disease patients and was not validated for SMA. Previous studies revealed normal quality of life and well-being in adult SMA patients even though these patients were physically severely disabled (26, 27). Such coping mechanisms possibly reduce the self-perception of NMS and thus might result in a difference between perceived and actual NMS. Based on a recent published study on health insurance datasets it can be assumed that NMS could be detected in increased rates if investigated by detailed diagnostic examination (9). However, datasets of health insurance are based on diagnoses made by physicians without the detailed knowledge how these have been made, thus are also biased. For example, general physical weakness and creatine kinase elevation might have been misinterpreted as a cardiac disease and not acknowledged as first symptoms of a yet undiagnosed SMA. Nevertheless, we cannot exclude that the frequency of some of the NMS mentioned above may in fact be higher in adult SMA patients, if it was ascertained either by a structured interview or by diagnostic procedures. Therefore, further studies to differ between perceived and actual NMS are needed.

## Data Availability Statement

The datasets generated for this study are available on request to the corresponding author.

## Ethics Statement

The studies involving human participants were reviewed and approved by by the local ethics committees of the participating centers. The patients/participants provided their written informed consent to participate in this study.

## Author Contributions

RG, CW, and AH designed and conceptualized the study. RG and AH drafted and wrote the manuscript. RG, CW, IC, JK, CK, DP, and EA had a major role in the acquisition of data. RG, EA, and AH analyzed the data and performed statistical analysis. All authors interpreted the data and critically revised the manuscript.

### Conflict of Interest

RG has received honoraria for presentations/advisory boards from Biogen. CW has received honoraria from Biogen as an advisory board member, for lectures and as a consultant from Hoffmann-La Roche. DP and EA report no disclosures. IC received a travel grant from Biogen. JK has received financial research support form TEVA Pharmaceuticals and honoraria as speaker/consultant for AbbVie, Ipsen, and AveXis/Novartis. CK has received honoraria from Biogen for presentations and as an advisory board member and for presentations from Merz Pharmaceuticals and Ipsen Pharmaceuticals. MD received a travel grant from Biogen and speaker honoraria from Desitin and Genzyme. PL has received financial research support from TEVA Pharmaceuticals and honoraria as speaker/consultant for AbbVie, BIAL, Desitin, Licher MT, Medtronic, Novartis. AL received financial research support from AB Science, Biogen Idec, Cytokinetics, GSK, Orion Pharam, Novartis, TauRx Therapeutics Ltd. and TEVA Pharmaceuticals. He also has received honoraria as a consultant from Mitsubishi, Orion Pharma, Novartis, Teva and as an advisory board member of Biogen, Treeway, and Hoffmann-La Roche. AH has received honoraria for presentations/advisory boards from Desitin and Biogen.
